# Some inequalities on the spectral radius of matrices

**DOI:** 10.1186/s13660-017-1598-2

**Published:** 2018-01-05

**Authors:** Linlin Zhao, Qingbing Liu

**Affiliations:** 1 College of Mathematics Science, Dezhou University, Dezhou, Shandong 253023 People’s Republic of China; 2 Department of Mathematics, Zhejiang Wanli University, Ningbo, Zhejiang 315100 People’s Republic of China

**Keywords:** 15A06, 15A42, 15B34, spectral radius, nonnegative matrix, Hadamard product

## Abstract

Let $A_{1}, A_{2},\ldots, A_{k}$ be nonnegative matrices. In this paper, some upper bounds for the spectral radius $\rho(A_{1}\circ A_{2}\circ\cdots\circ A_{k})$ are proposed. These bounds generalize some existing results, and comparisons between these bounds are also considered.

## Introduction

Let $M_{n}$ denote the set of all $n\times n$ complex matrices and $A=(a_{ij}), B=(b_{ij}) \in M_{n}$. If $a_{ij}-b_{ij}\geq0$, we say that $A\geq B$, and if $a_{ij} \geq0$, we say that *A* is nonnegative, denoted by $A\geq0$. The symbol $\rho(A)$ stands for the spectral radius of *A*. If *A* is a nonnegative matrix, the Perron-Frobenius theorem guarantees that $\rho(A)\in\sigma(A)$, where $\sigma(A)$ denotes the spectrum of *A*.

If there does not exist a permutation matrix *P* such that

$$P^{T}AP=\left ( \textstyle\begin{array}{@{}c@{\quad}c@{}} A_{1}&A_{12}\\ 0&A_{2} \end{array}\displaystyle \right ), $$

where $A_{1}$, $A_{2}$ are square matrices, then *A* is called irreducible.

Let *A* be an irreducible nonnegative matrix. It is well known that there exists a positive vector *u* such that $Au=\rho(A)u$, *u* being called a right Perron eigenvector of *A*.

The Hadamard product of *A*, *B* is defined as $A\circ B=(a_{ij}b_{ij})\in M_{n}$.

Let $A\geq0$, $B\geq0$. By using the Gersgorin theorem, Brauer theorem and Brualdi theorem, respectively, the authors of [1–5] have given some inequalities for the upper bounds of $\rho(A\circ B)$. Audenaert [6], Horn and Zhang [7] proved a beautiful inequality on $\rho(A\circ B)$ for nonnegative matrices *A* and *B*, that is, $\rho(A\circ B)\leq\rho(A B)$. Huang [8] generalized the above inequality to any *k* nonnegative matrices, that is, $\rho(A_{1}\circ A_{2}\circ\cdots\circ A_{k})\leq\rho(A_{1}A_{2}\cdots A_{k})$. Motivated by [8] and [1–4, 9, 10], in this paper we propose some inequalities on the upper bounds for the spectral radius of the Hadamard product of any *k* nonnegative matrices. These bounds generalize some existing results, and some comparisons between these bounds are also considered.

## Main results

First, we give some lemmas which are useful for obtaining the main results.

### Lemma 2.1

([11])

*Let* $A\in M_{n}$ *be a nonnegative matrix*. *If* $A_{k}$ *is a principal submatrix of* *A*, *then* $\rho(A_{k})\leq\rho(A)$. *If* *A* *is irreducible and* $A_{k}\neq A$, *then* $\rho(A_{k})< \rho(A)$.

### Lemma 2.2

([11])

*If* $A\in M_{n}$ *is an irreducible nonnegative matrix*, *and* $Az \leq kz$ *for a nonzero nonnegative vector* *z*, *then* $\rho(A)\leq k$.

### Lemma 2.3

([12])

*Let* $A = (a_{ij}) \in M_{n}$ *be a nonnegative matrix*. *Then*

$$ \rho(A)\leq\max_{ i\neq j} \frac{1}{2} \biggl\{ a_{ii}+a_{jj}+ \biggl[(a_{ii}-a_{jj})^{2}+4 \sum_{k\neq i}a_{ik}\sum _{k\neq j}a_{jk} \biggr]^{\frac{1}{2}} \biggr\} . $$

### Lemma 2.4

*Let* $A_{1}, A_{2},\ldots, A_{k} \in M_{n}$ *and* $D_{1}, D_{2}, \ldots , D_{k}$ *be diagonal matrices of order* *n*, *then*

$$\begin{aligned}& D^{-1}(A_{1}\circ A_{2}\circ\cdots\circ A_{k})D \\& \quad =\bigl(D_{1}^{-1}A_{1}D_{1}\bigr) \circ\bigl(D_{2}^{-1}A_{2}D_{2}\bigr)\circ \cdots\circ\bigl(D_{k}^{-1}A_{k}D_{k} \bigr), \end{aligned}$$

*where* *D* *equals the product of the matrices* $D_{k},D_{k-1},\ldots, D_{1}$, *that is*, $D=D_{k} \cdots D_{2}D_{1}$.

### Proof

Let $d_{r,i}$ be the *i*th diagonal of $D_{r}$ and $a_{r,ij}$ be the $(i,j)$ entry of $A_{r}$ ($r=1,2,\ldots, k$). Then the $(i,j)$ entry of $D^{-1}(A_{1}\circ A_{2}\circ\cdots\circ A_{k})D$ is

$$ \frac{1}{\prod_{r=1}^{k}d_{r,i}} \Biggl(\prod_{r=1}^{k}a_{r,ij} \Biggr)\prod_{r=1}^{k}d_{r,j}=\prod _{r=1}^{k} \biggl(\frac {1}{d_{r,i}}a_{r,ij}d_{r,j} \biggr), $$

which coincides with the $(i,j)$ entry of $(D_{1}^{-1}A_{1}D_{1})\circ (D_{2}^{-1}A_{2}D_{2})\circ\cdots\circ(D_{k}^{-1}A_{k}D_{k})$. The proof is completed. □

### Theorem 2.1

*Let* $A_{1}, A_{2}, \ldots,A_{k}\in M_{n}$ *and* $A_{1}=(a_{ij})\geq0, A_{2}=(b_{ij})\geq0, \ldots, A_{k}=(k_{ij})\geq 0$. *Then*

2.1 $$\begin{aligned}& \rho(A_{1}\circ A_{2}\circ\cdots\circ A_{k}) \\& \quad \leq\max_{1\leq i\leq n} \bigl\{ a_{ii}b_{ii} \cdots k_{ii}+\bigl(\rho(A_{1})-a_{ii}\bigr) \bigl( \rho(A_{2})-b_{ii}\bigr)\cdots \bigl(\rho(A_{k})-k_{ii} \bigr) \bigr\} . \end{aligned}$$

### Proof

If $A_{1}\circ A_{2}\circ\cdots\circ A_{k}$ is irreducible, then $A_{1},A_{2}, \ldots, A_{k}$ are all irreducible. From Lemma 2.1, we have

$$ \rho(A_{1})-a_{ii}>0,\qquad \rho(A_{2})-b_{ii}>0, \qquad \ldots,\qquad \rho(A_{k})-k_{ii}>0,\quad \forall i\in N. $$

Since $A_{1},A_{2},\ldots, A_{k}$ are nonnegative irreducible, there exist *k* positive vectors $u, v, \ldots, w$ such that $A_{1}u=\rho(A_{1})u, A_{2}^{T}v=\rho(A_{2})v, \ldots, A_{k}^{T}w=\rho(A_{k})w$. Thus, we have

$$\begin{aligned}& a_{ii}u_{i}+\sum_{j\neq i}a_{ij}u_{j}= \rho(A_{1})u_{i},\quad \forall i\in N, \\& b_{jj}v_{j}+\sum_{i\neq j}b_{ij}v_{i}= \rho(A_{2})v_{j},\quad \forall j\in N, \\& \ldots, \\& k_{jj}w_{j}+\sum_{i\neq j}k_{ij}w_{i}= \rho(A_{k})w_{j},\quad \forall j\in N. \end{aligned}$$

Thus, we have

$$ b_{ij}\leq\frac{ [\rho(A_{2})-b_{jj} ] v_{j} }{v_{i}}, \qquad \ldots,\qquad k_{ij}\leq \frac{ [\rho(A_{k})-k_{jj} ] w_{j} }{w_{i}}. $$

Let *z* be the vector $(z_{i})$, where

$$ z_{i}=\frac{u_{i}}{(\rho(A_{2})-b_{ii})v_{i} \cdots(\rho(A_{k})-k_{ii})w_{i}}>0,\quad \forall i\in N. $$

We define $P=A_{1}\circ A_{2}\circ\cdots\circ A_{k}$. For any $i\in N$,

$$\begin{aligned} (Pz)_{i} =&a_{ii}b_{ii}\cdots k_{ii}z_{i}+\sum_{i\neq j}a_{ij}b_{ij} \cdots k_{ij}z_{j} \\ \leq& a_{ii}b_{ii}\cdots k_{ii}z_{i}+ \sum_{i\neq j}a_{ij}\frac{(\rho (A_{2})-b_{jj}) v_{j} }{v_{i}} \cdots \frac{(\rho(A_{k})-k_{jj})w_{j} }{w_{i}}z_{j}. \end{aligned}$$

For

$$ z_{j}=\frac{u_{j}}{(\rho(A_{2})-b_{jj})v_{j} \cdots(\rho(A_{k})-k_{jj})w_{j}}, $$

we have

$$\begin{aligned} (Pz)_{i} \leq& a_{ii}b_{ii}\cdots k_{ii}z_{i}+\frac{1}{v_{i}\cdots w_{i}}\sum _{i\neq j}a_{ij}u_{j} \\ =&a_{ii}b_{ii}\cdots k_{ii}z_{i}+ \frac{1}{v_{i}\cdots w_{i}}\bigl(\rho (A_{1})-a_{ii} \bigr)u_{i} \\ =&a_{ii}b_{ii}\cdots k_{ii}z_{i}+ \bigl(\rho(A_{2})-b_{ii}\bigr) \cdots\bigl(\rho (A_{k})-k_{ii}\bigr) \bigl(\rho(A_{1})-a_{ii} \bigr)z_{i} \\ =&\bigl\{ a_{ii}b_{ii}\cdots k_{ii}+ \bigl( \rho(A_{1})-a_{ii}\bigr) \bigl(\rho(A_{2})-b_{ii} \bigr) \cdots\bigl(\rho(A_{k})-k_{ii}\bigr)\bigr\} z_{i}. \end{aligned}$$

By Lemma 2.2, this shows that

$$ \rho(A_{1}\circ\cdots\circ A_{k})\leq\max _{1\leq i\leq n} \bigl\{ a_{ii}b_{ii}\cdots k_{ii}+ \bigl(\rho(A_{1})-a_{ii}\bigr) \bigl( \rho(A_{2})-b_{ii}\bigr) \cdots\bigl(\rho(A_{k})-k_{ii} \bigr)\bigr\} . $$

If $A_{1}\circ A_{2}\circ\cdots\circ A_{k}$ is reducible, we denote by $P=(p_{ij})$ the $n\times n$ permutation matrix with $p_{12}=p_{23}=\cdots=p_{n1}=1$, the remaining $p_{ij}=0$, then all $A_{1}+tP, A_{2}+tP, \ldots, A_{k}+tP$ are nonnegative irreducible matrices for any chosen positive real numbers *t*. We substitute $A_{1}+tP, A_{2}+tP, \ldots , A_{k}+tP$ for $A_{1},A_{2},\ldots, A_{k}$, respectively, in the previous case, and then, letting $t\rightarrow0$, the result follows by continuity. The proof is completed. □

Setting $k=2$ in Theorem 2.1, we have the following corollary.

### Corollary 2.1

([1])

*Let* $A_{1}, A_{2}\in M_{n}$ *and* $A_{1}\geq0$, $A_{2}\geq0$. *Then*

$$ \rho(A_{1}\circ A_{2})\leq\max_{1\leq i\leq n} \bigl\{ a_{ii}b_{ii}+\bigl(\rho(A_{1})-a_{ii} \bigr) \bigl(\rho(A_{2})-b_{ii}\bigr) \bigr\} . $$

### Theorem 2.2

*Let* $A_{1}, A_{2}, \ldots,A_{k}\in M_{n}$ *and* $A_{1}=(a_{ij})\geq0, A_{2}=(b_{ij})\geq0, \ldots, A_{k}=(k_{ij})\geq 0$. *Then*

2.2 $$\begin{aligned}& \rho(A_{1}\circ A_{2}\circ\cdots\circ A_{k}) \\& \quad \leq\max_{ i\neq j} \frac{1}{2}\bigl\{ a_{ii}b_{ii}\cdots k_{ii}+a_{jj}b_{jj} \cdots k_{jj}+ \bigl[(a_{ii}b_{ii}\cdots k_{ii}-a_{jj}b_{jj}\cdots k_{jj})^{2} \\& \qquad {}+4\bigl(\rho(A_{1})-a_{ii}\bigr)\cdots\bigl( \rho(A_{k})-k_{ii}\bigr) \bigl(\rho (A_{1})-a_{jj} \bigr)\cdots\bigl(\rho(A_{k})-k_{jj}\bigr) \bigr]^{\frac{1}{2}}\bigr\} . \end{aligned}$$

### Proof

First we assume that $A_{1}\circ A_{2}\circ\cdots\circ A_{k}$ is irreducible. Obviously, $A_{1},A_{2}, \ldots, A_{k}$ are all irreducible, from Lemma 2.1, we have

$$ \rho(A_{1})-a_{ii}>0,\qquad \rho(A_{2})-b_{ii}>0, \qquad \ldots, \qquad \rho(A_{k})-k_{ii}>0,\quad \forall i\in N. $$

For the irreducibility of $A_{1},A_{2},\ldots, A_{k}$, there exist *k* positive vectors $u=(u_{i}), v=(v_{i}), \ldots, w=(w_{i})$ such that $A_{1}u=\rho(A_{1})u, A_{2}v=\rho(A_{2})v, \ldots, A_{k}w=\rho(A_{k})w$. Thus, we have

$$\begin{aligned}& a_{ii}+\sum_{j\neq i}\frac{a_{ij}u_{j}}{u_{i}}= \rho(A_{1}),\quad \forall i\in N, \\& b_{ii}+\sum_{j\neq i}\frac{b_{ij}v_{j}}{v_{i}}= \rho(A_{2}),\quad \forall i\in N, \\& \ldots, \\& k_{ii}+\sum_{j\neq i}\frac{k_{ij}w_{j}}{w_{i}}= \rho(A_{k}),\quad \forall i\in N. \end{aligned}$$

Define

$$\begin{aligned}& U=\operatorname{diag}(u_{1},u_{2},\ldots,u_{n}), \qquad V=\operatorname{diag}(v_{1},v_{2},\ldots,v_{n}), \qquad \ldots, \\& W=\operatorname{diag}(w_{1},w_{2}, \ldots,w_{n}). \end{aligned}$$

Let

$$\begin{aligned}& \hat{A}_{1}=(\hat{a}_{ij})=U^{-1}A_{1}U= \biggl(\frac{1}{u_{i}}a_{ij}u_{j} \biggr), \\& \hat{A}_{2}=(\hat{b}_{ij})=V^{-1}A_{2}V \biggl(\frac{1}{v_{i}}b_{ij}v_{j} \biggr), \\& \ldots, \\& \hat{A}_{k}=(\hat{k}_{ij})=W^{-1}A_{k}W \biggl(\frac{1}{w_{i}}k_{ij}w_{j} \biggr). \end{aligned}$$

It is easy to show that $\hat{A}_{1}, \hat{A}_{2}, \ldots, \hat{A}_{k}$ are all nonnegative irreducible matrices, and all the row sums of $\hat{A}_{1},\hat{A}_{2}, \ldots, \hat{A}_{k}$ are equal to $\rho(A_{1}),\rho(A_{2}),\ldots ,\rho(A_{k})$, respectively.

Let $D=W \cdots VU$ be the product of *k* nonsingular diagonal matrices $U, V, \ldots, W$. According to Lemma 2.4, we have

$$\begin{aligned}& D^{-1}(A_{1}\circ A_{2}\circ\cdots\circ A_{k})D \\& \quad =\bigl(U^{-1}A_{1}U\bigr)\circ\bigl(V^{-1}A_{2}V \bigr)\circ\cdots\circ\bigl(W^{-1}A_{k}W\bigr) \\& \quad =\hat{A}_{1} \circ\hat{A}_{2} \circ\cdots \circ\hat{A}_{k}. \end{aligned}$$

Thus, we have $\rho(A_{1}\circ A_{2}\circ\cdots\circ A_{k})=\rho(\hat{A}_{1}\circ\hat{A}_{2}\circ\cdots\circ\hat{A}_{k})$. From Lemma 2.3, we have

$$\begin{aligned}& \rho(\hat{A}_{1}\circ\hat{A}_{2}\circ\cdots\circ\hat{A}_{k}) \\& \quad \leq\max_{ i\neq j} \frac{1}{2}\biggl\{ \hat{a}_{ii} \hat{b}_{ii}\cdots \hat{k}_{ii}+\hat{a}_{jj}\hat{b}_{jj}\cdots\hat{k}_{jj}+ \biggl[(\hat{a}_{ii}\hat{b}_{ii}\cdots\hat{k}_{ii}-\hat{a}_{jj}\hat{b}_{jj}\cdots\hat{k}_{jj})^{2} \\& \qquad {}+4\biggl(\sum_{k\neq i}\hat{a}_{ik} \hat{b}_{ik} \cdots\hat{k}_{ik}\biggr) \biggl(\sum _{k\neq j}\hat{a}_{jk} \hat{b}_{jk} \cdots\hat{k}_{jk}\biggr) \biggr]^{\frac {1}{2}} \biggr\} \\& \quad \leq\max_{ i\neq j} \frac{1}{2}\biggl\{ a_{ii} b_{ii}\cdots k_{ii}+ a_{jj} b_{jj}\cdots k_{jj}+ \biggl[( a_{ii} b_{ii}\cdots k_{ii}- a_{jj} b_{jj}\cdots k_{jj})^{2} \\& \qquad {}+4\biggl(\sum_{k\neq i}\frac{a_{ik}u_{k}}{u_{i}} \sum _{k\neq i}\frac {b_{ik}v_{k}}{v_{i}} \cdots\sum _{k\neq i}\frac{ k_{ik}w_{k}}{w_{i}}\biggr) \biggl(\sum _{k\neq j}\frac{a_{jk}u_{k}}{u_{j}} \sum_{k\neq j} \frac{b_{ik}v_{k}}{v_{j}} \cdots\sum_{k\neq j}\frac{ k_{jk}w_{k}}{w_{j}} \biggr) \biggr]^{\frac{1}{2}} \biggr\} \\& \quad = \max_{ i\neq j} \frac{1}{2}\bigl\{ a_{ii} b_{ii}\cdots k_{ii}+ a_{jj} b_{jj}\cdots k_{jj}+ \bigl[( a_{ii} b_{ii}\cdots k_{ii}-a_{jj} b_{jj}\cdots k_{jj})^{2} \\& \qquad {}+4\bigl(\rho(A_{1})-a_{ii}\bigr) \bigl( \rho(A_{2})-b_{ii}\bigr)\cdots\bigl(\rho(A_{k})-k_{ii} \bigr) \bigl(\rho (A_{1})-a_{jj}\bigr) \\& \qquad {}\times{\bigl(\rho(A_{2})-b_{jj}\bigr)\cdots\bigl( \rho(A_{k})-k_{jj}\bigr)} \bigr]^{\frac {1}{2}} \bigr\} . \end{aligned}$$

If $A_{1}\circ A_{2}\circ\cdots\circ A_{k}$ is reducible, the proof is similar to Theorem 2.1. So, the proof is completed. □

Setting $k=2$ in Theorem 2.2, we have the following corollary.

### Corollary 2.2

([2])

*Let* $A_{1}, A_{2}\in M_{n}$ *and* $A_{1}\geq0$, $A_{2}\geq0$. *Then*

$$\begin{aligned} \rho(A_{1}\circ A_{2}) \leq&\max_{ i\neq j} \frac{1}{2}\bigl\{ a_{ii}b_{ii}+a_{jj}b_{jj}+ \bigl[(a_{ii}b_{ii}-a_{jj}b_{jj})^{2} \\ &{} +4\bigl(\rho(A_{1})-a_{ii}\bigr) \bigl( \rho(A_{2})-b_{ii}\bigr) \bigl(\rho(A_{1})-a_{jj} \bigr) \bigl(\rho (A_{2})-b_{jj}\bigr)\bigr]^{\frac{1}{2}} \bigr\} . \end{aligned}$$

We next give a simple comparison between the upper bound in (2.1) and the upper bound in (2.2). Without loss of generality, for $i\neq j$, assume that

$$\begin{aligned}& a_{ii}b_{ii}\cdots k_{ii}+\bigl( \rho(A_{1})-a_{ii}\bigr) \bigl(\rho(A_{2})-b_{ii} \bigr)\cdots \bigl(\rho(A_{k})-k_{ii}\bigr) \\& \quad \geq a_{jj}b_{jj} \cdots k_{jj}+\bigl( \rho(A_{1})-a_{jj}\bigr) \bigl(\rho (A_{2})-b_{jj} \bigr)\cdots\bigl(\rho(A_{k})-k_{jj}\bigr). \end{aligned}$$

Let $\gamma=a_{ii}b_{ii}\cdots k_{ii}+a_{jj}b_{jj}\cdots k_{jj}$. From (2.2), we have

$$\begin{aligned}& a_{ii}b_{ii}\cdots k_{ii}+a_{jj}b_{jj} \cdots k_{jj}+ \bigl[(a_{ii}b_{ii}\cdots k_{ii}-a_{jj}b_{jj}\cdots k_{jj})^{2} \\& \qquad {}+ 4\bigl(\rho(A_{1})-a_{ii}\bigr) \bigl( \rho(A_{2})-b_{ii}\bigr)\cdots\bigl(\rho(A_{k})-k_{ii} \bigr) \bigl(\rho (A_{1})-a_{jj}\bigr) \\& \qquad {}\times{ \bigl(\rho(A_{2})-b_{jj}\bigr)\cdots\bigl( \rho(A_{k})-k_{jj}\bigr)} \bigr]^{\frac {1}{2}} \\& \quad \leq\gamma+ \bigl\{ (a_{ii}b_{ii}\cdots k_{ii}-a_{jj}b_{jj}\cdots k_{jj})^{2}+ 4\bigl(\rho(A_{1})-a_{ii}\bigr)\cdots\bigl( \rho(A_{k})-k_{ii}\bigr) \\& \qquad {}\times\bigl[\bigl( \rho(A_{1})-a_{ii}\bigr) \bigl( \rho(A_{2})-b_{ii}\bigr)\cdots\bigl(\rho (A_{k})-k_{ii} \bigr)+a_{ii}b_{ii}\cdots k_{ii}-a_{jj}b_{jj} \cdots k_{jj} \bigr]\bigr\} ^{\frac{1}{2}} \\& \quad =\gamma+\bigl[ \bigl(a_{ii}b_{ii}\cdots k_{ii}-a_{jj}b_{jj}\cdots k_{jj} +2\bigl( \rho(A_{1})-a_{ii}\bigr)\cdots\bigl(\rho(A_{k})-k_{ii} \bigr)\bigr)^{2} \bigr]^{\frac{1}{2}} \\& \quad =2a_{ii}b_{ii}\cdots k_{ii}+2\bigl( \rho(A_{1})-a_{ii}\bigr) \bigl(\rho (A_{2})-b_{ii} \bigr)\cdots\bigl(\rho(A_{k})-k_{ii}\bigr). \end{aligned}$$

Thus, we have

$$\begin{aligned}& \rho(A_{1}\circ A_{2}\circ\cdots\circ A_{k}) \\& \quad \leq\max_{ i\neq j} \frac{1}{2}\bigl\{ a_{ii} \cdots k_{ii}+a_{jj}\cdots k_{jj}+ \bigl[(a_{ii}\cdots k_{ii}-a_{jj}\cdots k_{jj})^{2} +4\bigl(\rho(A_{1})-a_{ii} \bigr) \\& \qquad {}\times\bigl(\rho(A_{2})-b_{ii}\bigr)\cdots\bigl( \rho(A_{k})-k_{ii}\bigr) \bigl(\rho (A_{1})-a_{jj} \bigr) \bigl(\rho(A_{2})-b_{jj}\bigr)\cdots\bigl( \rho(A_{k})-k_{jj}\bigr)\bigr]^{\frac {1}{2}}\bigr\} \\& \quad \leq\max_{ 1 \leq i \leq n} \frac{1}{2} \bigl[2a_{ii}b_{ii} \cdots k_{ii}+2\bigl(\rho(A_{1})-a_{ii}\bigr)\cdots \bigl(\rho(A_{k})-k_{ii}\bigr) \bigr] \\& \quad =\max_{ 1 \leq i \leq n} \bigl[a_{ii}b_{ii}\cdots k_{ii}+\bigl(\rho (A_{1})-a_{ii}\bigr) \bigl( \rho(A_{2})-b_{ii}\bigr)\cdots\bigl(\rho(A_{k})-k_{ii} \bigr) \bigr]. \end{aligned}$$

Hence, bound (2.2) is better than bound (2.1).

In [8], the author proved that

2.3 $$ \rho(A_{1}\circ A_{2}\circ\cdots\circ A_{k})\leq\rho(A_{1}A_{2}\cdots A_{k}). $$

At present, we cannot give the comparison between bounds (2.1) and (2.3) or bounds (2.2) and (2.3), but the following numerical example shows that bounds (2.1) and (2.2) are better than (2.3). Next,we give an example: Consider four $4\times4$ nonnegative matrices

$$\begin{aligned}& A=\left ( \textstyle\begin{array}{@{}c@{\quad}c@{\quad}c@{\quad}c@{}} 4&1&0&2\\ 0& 0.05&1&1\\ 0& 0&4 &0.5\\ 1 & 0.5&0&4 \end{array}\displaystyle \right ),\qquad B=\left ( \textstyle\begin{array}{@{}c@{\quad}c@{\quad}c@{\quad}c@{}} 1&1&1&1\\ 1&1&1&1\\ 1&1&1&1\\ 1&1&1&1 \end{array}\displaystyle \right ), \\& C=\left ( \textstyle\begin{array}{@{}c@{\quad}c@{\quad}c@{\quad}c@{}} 2 &0&1&1\\ 1&4&0.5&0.5\\ 1&0&3&0.5\\ 0.5&1&1&2 \end{array}\displaystyle \right ),\qquad D=\left ( \textstyle\begin{array}{@{}c@{\quad}c@{\quad}c@{\quad}c@{}} 2 &0.5&0.5&0.5\\ 1&1&1&1\\ 0.5&0&2&0.5\\ 0&1&1&2 \end{array}\displaystyle \right ). \end{aligned}$$

(i) It is easy to calculate that $\rho(A\circ B)=5.4983$. By inequalities (2.1) and (2.2), we have

$$ \rho(A\circ B)\leq\max_{1\leq i\leq4 }\bigl\{ a_{ii}b_{ii}+ \bigl(\rho(A)-a_{ii}\bigr) \bigl(\rho(B)-b_{ii}\bigr)\bigr\} =16.3949, $$

and

$$\rho(A\circ B)\leq11.6478. $$

By inequality (2.3), we have

$$\rho(A\circ B)\leq\rho(AB)=19.05. $$

(ii) From calculation, we get $\rho(A\circ B\circ C)=12.0014$. By inequalities (2.1) and (2.2), we have

$$ \rho(A\circ B\circ C)\leq\max_{1\leq i\leq4 }\bigl\{ a_{ii}b_{ii}c_{ii}+ \bigl(\rho(A)-a_{ii}\bigr) \bigl(\rho(B)-b_{ii}\bigr) \bigl( \rho(C)-c_{ii}\bigr)\bigr\} =20.8846, $$

and

$$\rho(A\circ B\circ C) \leq17.8268. $$

By inequality (2.3), we have

$$\rho(A\circ B\circ C)\leq\rho(ABC)=88.5. $$

(iii) Let $A\circ B\circ C \circ D=G=(g_{ij})$. Then

$$ G=\left ( \textstyle\begin{array}{@{}c@{\quad}c@{\quad}c@{\quad}c@{}} 16 &0&0&1\\ 0&0.2&0.5&0.5\\ 0&0&24&0.075\\ 0&0.5&0&16 \end{array}\displaystyle \right ),\qquad ABCD=\left ( \textstyle\begin{array}{@{}c@{\quad}c@{\quad}c@{\quad}c@{}} 117.25 &78.75&155.75&126 \\ 34.3375 & 23.0625 & 45.6125 & 36.9\\ 75.375 & 50.625& 100.125 & 81\\ 92.125 & 61.875& 122.375 & 99 \end{array}\displaystyle \right ). $$

It is easy to calculate that $\rho(G)=24.0001$. By inequalities (2.1) and (2.2), we have

$$ \rho(G)\leq\max_{1\leq i\leq4} \bigl\{ a_{ii}b_{ii}c_{ii}d_{ii}+ \bigl(\rho(A)-a_{ii}\bigr) \bigl(\rho(B)-b_{ii}\bigr) \bigl( \rho (C)-c_{ii}\bigr) \bigl(\rho(D)-d_{ii}\bigr)\bigr\} =36.6608 $$

and

$$\begin{aligned} \rho(G) \leq&\max_{i\neq j} \frac{1}{2}\bigl\{ g_{ii}+g_{jj}+\bigl[(g_{ii}-g_{jj})^{2}+4 \bigl(\rho(A)-a_{ii}\bigr) \bigl(\rho (B)-b_{ii}\bigr) \bigl( \rho(C)-c_{ii}\bigr) \\ &{}\times\bigl(\rho(D)-d_{ii}\bigr) \bigl(\rho(A)-a_{jj} \bigr) \bigl(\rho(B)-b_{jj}\bigr) \bigl(\rho(C)-c_{jj}\bigr) \bigl(\rho (D)-d_{jj}\bigr)\bigr]^{\frac{1}{2}}\bigr\} \\ =&32.4451. \end{aligned}$$

By inequality (2.3), we have $\rho(G)\leq\rho(ABCD)=339.44$.

Next, we will give some other inequalities for $\rho(A_{1}\circ A_{2}\circ \cdots\circ A_{k})$. For $A_{1}\geq0$, write $L_{1}=A_{1}-\operatorname{diag}(a_{11}, \ldots,a_{nn})$. We denote $J_{A_{1}}=D_{1}^{-1}L_{1} $ with $D_{1}=\operatorname{diag}(d_{ii})$, where

$$d_{ii}= \textstyle\begin{cases} a_{ii},& \mbox{if } a_{ii}\neq0, \\ 1,& \mbox{if } a_{ii}= 0. \end{cases} $$

Then $J_{A_{1}}$ is nonnegative.

For $A_{2}\geq0$, let $D_{2}=\operatorname{diag}(s_{ii}), \ldots$ , for $A_{k} \geq0$, let $D_{k}=\operatorname{diag}(t_{ii})$ with

$$\begin{aligned}& s_{ii}= \textstyle\begin{cases} b_{ii},& \mbox{if } b_{ii}\neq0, \\ 1,& \mbox{if } b_{ii}= 0, \end{cases}\displaystyle \\& \ldots, \\& t_{ii}= \textstyle\begin{cases} k_{ii},& \mbox{if } k_{ii}\neq0, \\ 1,& \mbox{if } k_{ii}= 0, \end{cases}\displaystyle \end{aligned}$$

respectively. Then the nonnegative matrix $J_{A_{2}}, \ldots, J_{A_{k}}$ can be similarly defined.

### Theorem 2.3

*Let* $A_{1}, A_{2}, \ldots,A_{k}\in M_{n}$ *and* $A_{1}\geq0, A_{2}\geq0, \ldots, A_{k}\geq0$. *Then*

2.4 $$\begin{aligned}& \rho(A_{1}\circ A_{2}\circ\cdots\circ A_{k}) \\& \quad \leq\max_{1\leq i\leq n} \bigl\{ a_{ii}b_{ii} \cdots k_{ii}+d_{ii}\rho(J_{A_{1}})s_{ii} \rho(J_{A_{2}})\cdots t_{ii}\rho(J_{A_{k}}) \bigr\} . \end{aligned}$$

### Proof

Let $Q=A_{1}\circ A_{2}\circ\cdots\circ A_{k}$. First assume that *Q* is irreducible. Obviously $A_{1},A_{2}, \ldots, A_{k}$ are all irreducible, and then $J_{A_{1}}, J_{A_{2}}, \ldots, J_{A_{k}}$ are all nonnegative irreducible, so there exist *k* positive vectors $x, y, \ldots, h$ such that $J_{A_{1}}x=\rho(J_{A_{1}})x, J_{A_{2}}y=\rho(J_{A_{2}})y, \ldots, J_{A_{k}}h=\rho(J_{A_{k}})h$. So, we have

$$ \sum_{j\neq i}\frac{a_{ij}x_{j}}{x_{i}}=d_{ii} \rho(J_{A_{1}}),\qquad \sum_{j\neq i} \frac{b_{ij}y_{j}}{y_{i}}=s_{ii}\rho(J_{A_{2}}), \qquad \ldots,\qquad \sum_{j\neq i}\frac{k_{ij}h_{j}}{h_{i}}=t_{ii} \rho(J_{A_{k}}). $$

Now let $z=(z_{i})$ be the vector, where $z_{i}=(x_{i}y_{i}\cdots h_{i})>0$ for all *i*. For the irreducible nonnegative matrix *Q*, we have

$$\begin{aligned} (Qz)_{i} =&a_{ii}b_{ii}\cdots k_{ii}z_{i}+\sum_{i\neq j}a_{ij}b_{ij} \cdots k_{ij}z_{j} \\ \leq& a_{ii}b_{ii}\cdots k_{ii}z_{i}+ \biggl(\sum_{i\neq j}a_{ij}x_{j} \biggr) \biggl(\sum_{i\neq j}b_{ij}y_{j} \biggr)\cdots\biggl(\sum_{i\neq j}k_{ij}h_{j} \biggr) \\ =& a_{ii}b_{ii}\cdots k_{ii}z_{i}+ \bigl(d_{ii}x_{i}\rho(J_{A_{1}})\bigr) \bigl(s_{ii}y_{i}\rho (J_{A_{2}})\bigr)\cdots \bigl(t_{ii}h_{i}\rho(J_{A_{k}})\bigr) \\ =&\bigl\{ a_{ii}b_{ii}\cdots k_{ii}+ d_{ii}\rho(J_{A_{1}})s_{ii}\rho (J_{A_{2}}) \cdots t_{ii}\rho(J_{A_{k}})\bigr\} z_{i}. \end{aligned}$$

By Lemma 2.2, this shows that

$$ \rho(A_{1}\circ A_{2}\circ\cdots\circ A_{k})\leq \max_{1\leq i\leq n} \bigl\{ a_{ii}b_{ii}\cdots k_{ii}+ d_{ii}\rho(J_{A_{1}})s_{ii}\rho (J_{A_{2}})\cdots t_{ii}\rho(J_{A_{k}})\bigr\} . $$

The proof is completed. □

Setting $k=2$ in Theorem 2.3, we have the following corollary.

### Corollary 2.3

([4])

*Let* $A_{1}, A_{2}\in M_{n}$ *and* $A_{1}\geq0$, $A_{2}\geq0$. *Then*

$$ \rho(A_{1}\circ A_{2})\leq\max_{1\leq i\leq n} \bigl\{ a_{ii}b_{ii}+ d_{ii}\rho(J_{A_{1}})s_{ii} \rho(J_{A_{2}}) \bigr\} . $$

### Theorem 2.4

*Let* $A_{1}, A_{2}, \ldots,A_{k}\in M_{n}$ *and* $A_{1}\geq0, A_{2}\geq0, \ldots, A_{k}\geq0$. *Then*

2.5 $$\begin{aligned}& \rho(A_{1}\circ A_{2}\circ\cdots\circ A_{k}) \\& \quad \leq\max_{ i\neq j} \frac{1}{2}\bigl\{ a_{ii}b_{ii}\cdots k_{ii}+a_{jj}b_{jj} \cdots k_{jj}+ \bigl[(a_{ii}b_{ii}\cdots k_{ii}-a_{jj}b_{jj}\cdots k_{jj})^{2} \\& \qquad {}+4 (d_{ii}s_{ii}\cdots t_{ii}) (d_{jj}s_{jj}\cdots t_{jj}) \bigl( \rho^{2}(J_{A_{1}})\rho^{2}(J_{A_{2}})\cdots \rho^{2}(J_{A_{k}})\bigr) \bigr]^{\frac{1}{2}}\bigr\} . \end{aligned}$$

### Proof

First we assume that $A_{1}\circ A_{2}\circ\cdots\circ A_{k}$ is irreducible. Obviously, $J_{A_{1}}, J_{A_{2}}, \ldots, J_{A_{k}}$ are all nonnegative irreducible, then there exist *k* positive vectors $x, y, \ldots, h$ such that $J_{A_{1}}x=\rho(J_{A_{1}})x, J_{A_{2}}y=\rho(J_{A_{2}})y, \ldots, J_{A_{k}}h=\rho(J_{A_{k}})h$. Thus, we have

$$ \sum_{j\neq i}\frac{a_{ij}x_{j}}{x_{i}}=d_{ii} \rho(J_{A_{1}}), \qquad \sum_{j\neq i} \frac{b_{ij}y_{j}}{y_{i}}=s_{ii}\rho(J_{A_{2}}), \qquad \ldots,\qquad \sum_{j\neq i}\frac{k_{ij}h_{j}}{h_{i}}=t_{ii} \rho(J_{A_{k}}). $$

Define

$$\begin{aligned}& X=\operatorname{diag}(x_{1},x_{2},\ldots,x_{n}), \qquad Y=\operatorname{diag}(y_{1},y_{2},\ldots,y_{n}), \qquad \ldots, \\& H=\operatorname{diag}(h_{1},h_{2}, \ldots,h_{n}). \end{aligned}$$

Let

$$ \tilde{A_{1}}=(\tilde{a}_{ij})=X^{-1}A_{1}X,\qquad \tilde{A_{2}}=(\tilde {b}_{ij})=Y^{-1}A_{2}Y,\qquad \ldots, \qquad \tilde{A_{k}}=(\tilde{k}_{ij})=H^{-1}A_{k}H. $$

From Lemma 2.4, we have

$$\begin{aligned}& \bigl(X^{-1}Y^{-1}\cdots H^{-1}\bigr) (A_{1}\circ A_{2}\circ\cdots\circ A_{k}) (H \cdots YX) \\& \quad =\bigl(X^{-1}A_{1}X\bigr)\circ\bigl(Y^{-1}A_{2}Y \bigr)\circ\cdots\circ\bigl(H^{-1}A_{k}H\bigr) \\& \quad =\tilde{A_{1}} \circ\tilde{A_{2}} \circ\cdots \circ \tilde{A_{k}}. \end{aligned}$$

Thus, $\rho(A_{1}\circ A_{2}\circ\cdots\circ A_{k})=\rho(\tilde{A_{1}} \circ \tilde{A_{2}} \circ\cdots\circ\tilde{A_{k}} )$. From Lemma 2.3, we have

$$\begin{aligned} \begin{aligned} &\rho(\tilde{A_{1}} \circ\tilde{A_{2}} \circ\cdots\circ \tilde{A_{k}}) \\ &\quad \leq\max_{ i\neq j} \frac{1}{2}\biggl\{ a_{ii} b_{ii}\cdots k_{ii}+ a_{jj} b_{jj}\cdots k_{jj}+ \biggl[( a_{ii} b_{ii}\cdots k_{ii}- a_{jj} b_{jj}\cdots k_{jj})^{2} \\ &\qquad {}+4\biggl(\sum_{k\neq i}\frac{a_{ik}x_{k}}{x_{i}} \frac{b_{ik}y_{k}}{y_{i}} \cdots \frac{ k_{ik}h_{k}}{h_{i}}\biggr) \biggl(\sum _{k\neq j}\frac{a_{jk}x_{k}}{x_{j}} \frac {b_{ik}y_{k}}{y_{j}} \cdots \frac{ k_{jk}h_{k}}{h_{j}}\biggr) \biggr]^{\frac {1}{2}} \biggr\} \\ &\quad \leq\max_{ i\neq j} \frac{1}{2}\biggl\{ a_{ii} b_{ii}\cdots k_{ii}+ a_{jj} b_{jj}\cdots k_{jj}+ \biggl[( a_{ii} b_{ii}\cdots k_{ii}- a_{jj} b_{jj}\cdots k_{jj})^{2} \\ &\qquad {}+4\biggl(\sum_{k\neq i}\frac{a_{ik}x_{k}}{x_{i}} \sum _{k\neq i}\frac {b_{ik}y_{k}}{y_{i}} \cdots\sum _{k\neq i}\frac{ k_{ik}h_{k}}{h_{i}}\biggr) \biggl(\sum _{k\neq j}\frac{a_{jk}x_{k}}{x_{j}} \sum_{k\neq j} \frac{b_{ik}y_{k}}{y_{j}} \cdots\sum_{k\neq j}\frac{ k_{jk}h_{k}}{h_{j}} \biggr) \biggr]^{\frac{1}{2}} \biggr\} \\ &\quad = \max_{ i\neq j} \frac{1}{2}\bigl\{ a_{ii} b_{ii}\cdots k_{ii}+ a_{jj}b_{jj}\cdots k_{jj}+ \bigl[( a_{ii} b_{ii}\cdots k_{ii}- a_{jj} b_{jj}\cdots k_{jj})^{2} \\ &\qquad {}+4(d_{ii}s_{ii}\cdots t_{ii}) (d_{jj}s_{jj}\cdots t_{jj}) \bigl(\rho ^{2}(J_{A_{1}})\rho^{2}(J_{A_{2}})\cdots \rho^{2}(J_{A_{k}})\bigr) \bigr]^{\frac {1}{2}} \bigr\} . \end{aligned} \end{aligned}$$

If $A_{1}\circ A_{2}\circ\cdots\circ A_{k}$ is reducible, then substituting $A_{1}+tP, A_{2}+tP, \ldots, A_{k}+tP$ for $A_{1},A_{2}, \ldots, A_{k}$, respectively, in the previous case, letting $t\rightarrow0$, the result is derived. □

Setting $k=2$ in Theorem 2.4, we have the following corollary.

### Corollary 2.4

([1])

*Let* $A_{1}, A_{2}\in M_{n}$ *and* $A_{1}\geq0$, $A_{2}\geq0$. *Then*

$$\rho(A_{1}\circ A_{2})\leq\max_{ i\neq j} \frac{1}{2} \bigl\{ a_{ii}b_{ii}+a_{jj}b_{jj}+ \bigl[(a_{ii}b_{ii}-a_{jj}b_{jj})^{2}+4d_{ii}s_{ii}d_{jj}s_{jj} \rho ^{2}(J_{A_{1}})\rho^{2}(J_{A_{2}}) \bigr]^{\frac{1}{2}} \bigr\} . $$

We next give a comparison between the upper bound in (2.4) and the upper bound in (2.5). Without loss of generality, for $i\neq j$, assume that

$$\begin{aligned}& a_{ii}b_{ii}\cdots k_{ii}+d_{ii}s_{ii} \cdots t_{ii} \rho (J_{A_{1}})\rho(J_{A_{2}})\cdots \rho(J_{A_{k}}) \\& \quad \geq a_{jj}b_{jj} \cdots k_{jj}+d_{jj}s_{jj} \cdots t_{jj} \rho (J_{A_{1}})\rho(J_{A_{2}})\cdots \rho(J_{A_{k}}). \end{aligned}$$

Let $\gamma=a_{ii}b_{ii}\cdots k_{ii}+a_{jj}b_{jj}\cdots k_{jj}$. From (2.5), we have

$$\begin{aligned}& a_{ii}b_{ii}\cdots k_{ii}+a_{jj}b_{jj} \cdots k_{jj}+ \bigl[(a_{ii}b_{ii}\cdots k_{ii}-a_{jj}b_{jj}\cdots k_{jj})^{2} \\& \qquad {}+4(d_{ii}s_{ii}\cdots t_{ii}) (d_{jj}s_{jj}\cdots t_{jj}) \bigl(\rho ^{2}(J_{A_{1}})\rho^{2}(J_{A_{2}})\cdots \rho^{2}(J_{A_{k}})\bigr) \bigr]^{\frac {1}{2}} \\& \quad \leq\gamma+ \bigl\{ (a_{ii}b_{ii}\cdots k_{ii}-a_{jj}b_{jj}\cdots k_{jj})^{2}+ 4d_{ii}s_{ii}\cdots t_{ii}\rho(J_{A_{1}}) \rho(J_{A_{2}})\cdots \rho(J_{A_{k}}) \\& \qquad {}\times\bigl[d_{ii}s_{ii}\cdots t_{ii} \rho(J_{A_{1}})\rho (J_{A_{2}})\cdots\rho(J_{A_{k}})+a_{ii}b_{ii} \cdots k_{ii}-a_{jj}b_{jj}\cdots k_{jj} \bigr]\bigr\} ^{\frac{1}{2}} \\& \quad =\gamma+\bigl[ \bigl(a_{ii}b_{ii}\cdots k_{ii}-a_{jj}b_{jj}\cdots k_{jj} +2d_{ii}s_{ii}\cdots t_{ii}\rho(J_{A_{1}}) \rho(J_{A_{2}})\cdots\rho (J_{A_{k}})\bigr)^{2} \bigr]^{\frac{1}{2}} \\& \quad =2a_{ii}b_{ii}\cdots k_{ii}+2d_{ii}s_{ii} \cdots t_{ii}\rho (J_{A_{1}})\rho(J_{A_{2}})\cdots \rho(J_{A_{k}}). \end{aligned}$$

Thus, from (2.5) and the above inequality, we have

$$\begin{aligned}& \rho(A_{1}\circ A_{2}\circ\cdots\circ A_{k}) \\& \quad \leq\max_{ i\neq j} \frac{1}{2}\bigl\{ a_{ii}b_{ii}\cdots k_{ii}+a_{jj}b_{jj} \cdots k_{jj}+ \bigl[(a_{ii}b_{ii}\cdots k_{ii}-a_{jj}b_{jj}\cdots k_{jj})^{2} \\& \qquad {}+4(d_{ii}s_{ii}\cdots t_{ii}) (d_{jj}s_{jj}\cdots t_{jj}) \bigl( \rho^{2}(J_{A_{1}})\rho^{2}(J_{A_{2}})\cdots \rho^{2}(J_{A_{k}})\bigr)\bigr]^{\frac{1}{2}}\bigr\} \\& \quad \leq\max_{ 1 \leq i\leq n} \frac{1}{2}\bigl(2a_{ii}b_{ii} \cdots k_{ii}+2d_{ii}s_{ii}\cdots t_{ii} \rho(J_{A_{1}})\rho(J_{A_{2}})\cdots\rho (J_{A_{k}})\bigr) \\& \quad =\max_{ 1 \leq i \leq n} \bigl(a_{ii}b_{ii}\cdots k_{ii}+d_{ii}s_{ii}\cdots t_{ii} \rho(J_{A_{1}})\rho(J_{A_{2}})\cdots\rho (J_{A_{k}})\bigr). \end{aligned}$$

Hence, the upper bound (2.5) is better than bound (2.4). Here too, we could not give the comparison between (2.4) and (2.3) or (2.5) and (2.3). Next, we give an example which shows that the results obtained in Theorems 2.3 and 2.4 are better than inequalities (2.3).

Let

$$\begin{aligned}& A=\left ( \textstyle\begin{array}{@{}c@{\quad}c@{\quad}c@{\quad}c@{}} 2 &0&1&1\\ 1&4&0.5&0.5\\ 1&0&3&0.5\\ 0.5&1&1&2 \end{array}\displaystyle \right ), \qquad B=\left ( \textstyle\begin{array}{@{}c@{\quad}c@{\quad}c@{\quad}c@{}} 2 &0.5&0.5&0.5\\ 1&1&1&1\\ 0.5&0&2&0.5\\ 0&1&1&2 \end{array}\displaystyle \right ), \\& C=\left ( \textstyle\begin{array}{@{}c@{\quad}c@{\quad}c@{\quad}c@{}} 4 &1&1&1\\ 2&2&1&1\\ 0&2&2&1\\ 1&1&1&1 \end{array}\displaystyle \right ),\qquad D=\left ( \textstyle\begin{array}{@{}c@{\quad}c@{\quad}c@{\quad}c@{}} 1 &0.5&2&0.5\\ 0.5&1&0.5&0\\ 0&0.5&1&0.5\\ 0&1&0.5&1 \end{array}\displaystyle \right ). \end{aligned}$$

Let $A\circ B\circ C \circ D=P=(p_{ij})$. Then

$$ P=\left ( \textstyle\begin{array}{@{}c@{\quad}c@{\quad}c@{\quad}c@{}} 16 &0&1&0.25\\ 1&8&0.25&0\\ 0&0&12&0.075\\ 0&1&0.5&4 \end{array}\displaystyle \right ),\qquad ABCD=\left ( \textstyle\begin{array}{@{}c@{\quad}c@{\quad}c@{\quad}c@{}} 35.5 &55.75 & 86 & 35.75\\ 57.75 & 91.875 & 139 & 57.875\\ 30.25 & 57.25 & 78 & 34.75\\ 34.875 &64.125 & 87.5 &38.875 \end{array}\displaystyle \right ). $$

It is easy to calculate that $\rho(P)=16.0028$. By inequalities (2.4) and (2.5), we have

$$ \rho(P)\leq\max_{1\leq i\leq4} \bigl\{ p_{ii}\bigl(1+ \rho(J_{A})\rho(J_{B})\rho(J_{C}) \rho(J_{D})\bigr)\bigr\} =36.2262 $$

and

$$\begin{aligned} \rho(P) \leq&\max_{i\neq j} \frac{1}{2}\bigl\{ p_{ii}+p_{jj}+\bigl[(p_{ii}-p_{jj})^{2}+4p_{ii}p_{jj} \rho ^{2}(J_{A})\rho^{2}(J_{B}) \rho^{2}(J_{C})\rho^{2}(J_{D}) \bigr]^{\frac{1}{2}}\bigr\} \\ =&29.6605. \end{aligned}$$

By inequality (2.3) and Lemma 2.1, we have $\rho(P)\leq91.875$.

## Conclusions

In this paper, we have proposed some upper bounds for $\rho(A_{1}\circ A_{2}\circ\cdots\circ A_{k})$ of the Hadamard product of matrices. These bounds generalize some corresponding results of [1–4].
